# Generating Extended Resolution Proofs with a BDD-Based SAT Solver

**DOI:** 10.1007/978-3-030-72016-2_5

**Published:** 2021-03-01

**Authors:** Randal E. Bryant, Marijn J. H. Heule

**Affiliations:** 8 Eindhoven University of Technology, Eindhoven, The Netherlands; 9 Aalborg University, Aalborg East, Denmark; Computer Science Department, Carnegie Mellon University, Pittsburgh, PA United States

**Keywords:** extended resolution, binary decision diagrams, mutilated chessboard, pigeonhole problem

## Abstract

In 2006, Biere, Jussila, and Sinz made the key observation that the underlying logic behind algorithms for constructing Reduced, Ordered Binary Decision Diagrams (BDDs) can be encoded as steps in a proof in the *extended resolution* logical framework. Through this, a BDD-based Boolean satisfiability (SAT) solver can generate a checkable proof of unsatisfiability. Such proofs indicate that the formula is truly unsatisfiable without requiring the user to trust the BDD package or the SAT solver built on top of it.

We extend their work to enable arbitrary existential quantification of the formula variables, a critical capability for BDD-based SAT solvers. We demonstrate the utility of this approach by applying a prototype solver to obtain polynomially sized proofs on benchmarks for the mutilated chessboard and pigeonhole problems—ones that are very challenging for search-based SAT solvers.

## 1 Introduction

When a Boolean satisfiability (SAT) solver returns a purported solution to a Boolean formula, its validity can easily be checked by making sure that the solution indeed satisfies the formula. When the formula is unsatisfiable, on the other hand, having the solver simply declare this to be the case requires the user to have faith in the solver, a complex piece of software that could well be flawed. Indeed, modern solvers employ a number of sophisticated techniques to reduce the search space. If one of those techniques is invalid or incorrectly implemented, the solver may overlook actual solutions and label a formula as unsatisfiable, even when it is not.

With SAT solvers providing the foundation for a number of different real-world tasks, this "false negative" outcome could have unacceptable consequences. For example, when used as part of a formal verification system, the usual strategy is to encode some undesired property of the system as a formula. The SAT solver is then used to determine whether some operation of the system could lead to this undesirable property. Having the solver declare the formula to be unsatisfiable is an indication that the undesirable behavior cannot occur, but only if the formula is truly unsatisfiable.

* Supported by the National Science Foundation under grant CCF-2010951

Rather than requiring users to place their trust in a complex software system, a *proof-generating* solver constructs a proof that the formula is indeed unsatisfiable. The proof has a form that can readily be checked by a simple proof checker. Initial work of checking unsatisfiability results was based on resolution proofs, but modern checkers are based on stronger proof systems [16,33]. The checker provides an independent validation that the formula is indeed unsatisfiable. The checker can even be simple enough to be formally verified [9,23,29]. Such a capability has become an essential feature for modern SAT solvers.

In their 2006 papers [21,28], Jussila, Sinz and Biere made the key observation that the underlying logic behind algorithms for constructing Reduced, Ordered Binary Decision Diagrams (BDDs) [4] can be encoded as steps in a proof in the *extended resolution* logical framework [30]. Through this, a BDD-based Boolean satisfiability solver can generate checkable proofs of unsatisfiability for a set of clauses. Such proofs indicate that the formula is truly unsatisfiable without requiring the user to trust the BDD package or the SAT solver built on top of it.

In this paper, we refine these ideas to enable a full-featured, BDD-based SAT solver. Chief among these is the ability to perform existential quantification on arbitrary variables. (Jussila, Sinz, and Biere [21] extended their original work [28] to allow existential quantification, but only for the root variable of a BDD.) In addition, we allow greater flexibility in the choice of variable ordering and the order in which conjunction and quantification operations are performed. This combination allows a wide range of strategies for creating a sequence of BDD operations that, starting with a set of input clauses, yield the BDD representation of the constant function **0**, indicating that the formula is unsatisfiable. Using the extended-resolution proof framework, these operations can generate a proof showing that the original set of clauses logically implies the empty clause, providing a checkable proof that the formula is unsatisfiable.

As the experimental results demonstrate, our refinements enable a proof-generating BDD-based SAT solver to achieve polynomial performance on several classic "hard" problems [1,15]. Since the performance of a proof-generating SAT solver affects not only the runtime of the program, but also the length of the proofs generated, achieving polynomial performance is an important step forward. Our results for these benchmarks rely on a novel approach to ordering the conjunction and quantification operations, inspired by symbolic model checking [7].

This paper is structured as follows. First, it provides a brief introduction to the resolution and extended resolution logical frameworks and to BDDs. Then we show how a BDD-based SAT solver can generate proofs by augmenting algorithms for computing the conjunction of two functions represented as BDDs, and for checking that one function logically implies another. We then describe our prototype implementation and evaluate its performance on several classic problems. We conclude with some general observations and suggestions for further work.

## 2 Preliminaries

Given a Boolean formula over a set of variables $\{x_1, x_2, \ldots, x_n\}$, a SAT solver attempts to find an assignment to these variables that will satisfy the formula, or it declares that the formula is unsatisfiable. As is standard practice, we use the term *literal* to refer to either a variable or its complement. Most SAT solvers use Boolean formulas expressed in *conjunctive normal form*, where the formula consists of a set of *clauses*, each consisting of a set of literals. Each clause is a disjunction: if an assignment sets any of its literals to true, the clause is considered to be satisfied. The overall formula is a conjunction: a satisfying assignment must satisfy all of the clauses.

We write $\top$ to denote both tautology and logical truth, and $\bot$ to represent both an empty clause and logical falsehood. When writing clauses, we omit disjunction symbols and use overlines to denote negation, writing $\overline{u} \vee v \vee \overline{w}$ as $\overline{u}\, v\, \overline{w}$.

### 2.1 (Extended) Resolution Proofs

Robinson [26] observed that a single inference rule could form the basis for a refutation theorem-proving technique for first-order logic. Here, we consider its specialization to propositional logic. For clauses of the form $C \vee x$, and $\overline{x} \vee D$, the resolution rule derives the new clause $C \vee D$. This inference is written with a notation showing the required conditions above a horizontal line, and the resulting inference (the *resolvent*) below:

$$\frac{C \vee x \qquad \overline{x} \vee D}{C \vee D}$$

Resolution provides a mechanism for proving that a set of clauses is unsatisfiable. Suppose the input consists of $m$ clauses. A resolution proof is given as a *trace* consisting of a series of *steps* $S$, where each step $s_i$ consists of a clause $C_i$ and a (possibly empty) list of antecedents $A_i$, where each antecedent is the index of one of the previous steps. The first set of steps, denoted $S_m$, consists of the input clauses without any antecedents. Each successive step then consists of a clause and a set of antecedents, such that the clause can be derived from the clauses in the antecedents by one or more resolution steps. It follows by transitivity that for each step $s_i$, with $i > m$, clause $C_i$ is logically implied by the input clauses, written $S_m \vdash C_i$. If, through a series of steps, we can reach a step $s_t$ where $C_t$ is the empty clause, then the trace provides a proof that $S_m \vdash \bot$, i.e., the set of input clauses is not satisfiable.

Tseitin [30] introduced the extended-resolution proof framework in 1966. It allows the addition of new *extension* variables to a resolution proof in a manner that preserves the integrity of the proof. In particular, in introducing variable $e$, there must be an accompanying set of clauses that encode $e \leftrightarrow F$, where $F$ is a formula over variables (both original and extension) that were introduced earlier. These are referred to as the *defining clauses* for extension variable $e$. Variable $e$ then provides a shorthand notation by which $F$ can be referenced multiple times. Doing so can reduce the size of a clausal representation of a problem by an exponential factor.

An extension variable $e$ is introduced into the proof by including its defining clauses in the list of clauses being generated. The proof checker must ensure that these added clauses do not artificially restrict the set of satisfying solutions. The checker can do this by making sure that the defining clauses are *blocked* with respect to variable $e$ [22]. That is, for each defining clause $C$ containing literal $e$ and each defining clause $D$ containing literal $\overline{e}$, there must be some literal $l$ in $C$ such that its complement $\overline{l}$ is in $D$. As a result, resolving clauses $C$ and $D$ will yield a tautology.

Tseitin transformations are commonly used to encode a logic circuit or formula as a set of clauses without requiring the formulas to be “flattened” into a conjunctive normal form over the circuit inputs or formula variables. These introduced variables are called *Tseitin variables* and are considered to be part of the input formula. An extended resolution proof takes this concept further by introducing additional variables as part of the proof. Some problems for which the minimum resolution proof must be of exponential size can be expressed with polynomial-sized proofs in extended resolution [8].

To validate the proofs, we use a clausal proof system, known as Resolution Asymmetric Tautology (RAT), that generalizes extended resolution [32]. RAT is used in industry and to validate the results of the SAT competitions [18]. There are various fast and formally-verified RAT proof checkers [10,23,29].

Clausal proofs also allow the removal of clauses. In our use, we delete clauses when the program can determine that they will not be referenced as antecedents for any succeeding clauses. As the experimental results of Section 4 demonstrate, deleting clauses that are no longer needed can substantially reduce the number of clauses the checker must track while processing a proof.

### 2.2 Binary Decision Diagrams

Reduced, Ordered Binary Decision Diagrams (which we refer to as simply “BDDs”) provide a canonical form for representing Boolean functions, and an associated set of algorithms for constructing them and testing their properties. A number of tutorials have been published [2,5,6]. providing a background on BDDs and their algorithms.

With BDDs, functions are defined over a set of variables $X = \{x_1, x_2, \ldots, x_n\}$. We let $L_1$ and $L_0$ denote the two leaf nodes, representing the constant functions **1** and **0**, respectively. Each nonterminal node $u$ has an associated variable $\mathsf{Var}(u)$ and children $\mathsf{Hi}(u)$, indicating the case where the node variable has value 1, and $\mathsf{Lo}(u)$, indicating the case where the node variable has value 0.

Nodes are stored in a *unique table*, indexed by the key $\langle \mathsf{Var}(u), \mathsf{Hi}(u), \mathsf{Lo}(u)\rangle$, so that isomorphic nodes are never created. The nodes are shared across all of the generated BDDs [24]. In presenting algorithms, we assume a function $\textsc{GetNode}(x, u_1, u_0)$ that checks the unique table for a node with variable $x$ and children $u_1$ and $u_0$. It either returns the node stored there, or it creates a new node and enters it into the table. With this table, we can guarantee that the subgraphs with root nodes $u$ and $v$ represent the same Boolean function if and only if $u = v$. We can therefore identify Boolean functions with their BDD root nodes.

BDD packages support multiple operations for constructing and testing the properties of Boolean functions represented by BDDs. A number of these are based on the *Apply* algorithm [4]. Given BDDs $u$ and $v$ representing functions $f$ and $g$, respectively, and a Boolean operation (e.g., AND), the algorithm generates the BDD representation $w$ of the operation applied to those functions (e.g., $f \wedge g$.) For each operation, the program maintains an *operation cache* indexed by the argument nodes $u$ and $v$, mapping to the result node $w$. With this cache, the worst case number of recursive steps required by the algorithm is bounded by the product of the sizes (in nodes) of the arguments.

We use the term APPLYAND to refer to the Apply algorithm for Boolean operation $\wedge$ and APPLYOR to refer to the Apply algorithm for Boolean operation $\vee$.

## 3 Proof Generation During BDD Construction

In our formulation, every newly created BDD node $u$ is assigned an extension variable. (As notation, we use the same name for the node and for its extension variable.) We then extend the Apply algorithm to generate proofs based on the recursive structure of the BDD operations.

Let $S_m$ denote the set of input clauses. Our goal is to generate a proof that $S_m \vdash \bot$, i.e., there is no satisfying assignment for these clauses. Our BDD-based approach generates a sequence of BDDs with root nodes $u_1, u_2, \ldots, u_t$, where $u_t = L_0$, based on a combination of the following operations. (The exact sequencing of operations is determined by the *evaluation mechanism*, as is described in Section 4.)

1. For input clause $C_i$ generate its BDD representation $u_i$ using a series of ApplyOr operations.
2. For roots $u_j$ and $u_k$, generate the BDD representation of their conjunction $u_l = u_j \wedge u_k$ using the ApplyAnd operation.
3. For root $u_j$ and some set of variables $Y \subseteq X$, perform existential quantification: $u_k = \exists Y\, u_j$.

Although the existential quantification operation is not mandatory for a BDD-based SAT solver, it can greatly improve its performance [13]. It is the BDD counterpart to Davis-Putnam variable elimination on clauses [11]. As the notation indicates, there are often multiple variables that can be eliminated simultaneously. Although the operation can cause a BDD to increase in size, it generally causes a reduction. Our experimental results demonstrate the importance of this operation.

As these operations proceed, we simultaneously generate a set of proof steps. The details of each step are given later in the presentation. For each BDD generated, we maintain the proof invariant that its root node $u_j$ satisfies $S_m \vdash u_j$.

1. Following the generation of the BDD $u_i$ for clause $C_i$, we also generate a proof that $C_i \vdash u_i$. This is described in Section 3.1.
2. Justifying the conjunctions requires two parts:
   (a) Using a modified version of the ApplyAnd algorithm we follow the structure of its recursive calls to generate a proof that the algorithm preserves implication: $u_j \wedge u_k \rightarrow u_l$. This is described in Section 3.2.
   (b) This implication can be combined with the earlier proofs that $S_m \vdash u_j$ and $S_m \vdash u_k$ to prove $S_m \vdash u_l$.
3. Justifying the quantification also requires two parts:
   (a) Following the generation of $u_k$ via existential quantification, we perform a separate check that $u_j \rightarrow u_k$. This check uses a proof-generating version of the Apply algorithm for implication testing that we refer to as ProveImplication. This is described in Section 3.3.
   (b) This implication can be combined with the earlier proof that $S_m \vdash u_j$ to prove $S_m \vdash u_k$.

As case 3(a) states, we do not attempt to track the detailed logic underlying the quantification operation. Instead, we run a separate check that the quantification preserves implication. As is the case with many BDD packages, our implementation can perform existential quantification of an arbitrary set of variables in a single pass over the argument BDD. A single implication test suffices for the entire quantification.

Sinz and Biere's formulation of proof generation by a BDD-based SAT solver [28] introduces special extension variables $n_1$ and $n_0$ to represent the BDD leaves $L_1$ and $L_0$. Their proof then includes unit clauses $n_1$ and $\overline{n}_0$ to force these variables to be set to 1 and 0, respectively. This formulation greatly reduces the number of special cases to consider in the proof-generating version of the ApplyAnd operation, but it complicates the generation of resolution proofs for the implication test. Instead, we directly associate leaves $L_1$ and $L_0$ with $\top$ and $\bot$, respectively.

The $n$ variables in the input clauses all have associated BDD variables. The proof then introduces an extension variable every time a new BDD node is created. In the following presentation, we use the node name (e.g., $u$) to indicate the associated extension variable. In the actual implementation, the extension variable identifier (an integer) is stored as one of the fields in the node representation.

When creating a new node, the GetNode function adds (up to) four defining clauses for the associated extension variable. For node $u$ with variable $\mathsf{Var}(u) = x$, $\mathsf{Hi}(u) = u_1$, and $\mathsf{Lo}(u) = u_0$, the clauses are:

| Notation | Formula | Clause |
|---|---|---|
| $\mathsf{HD}(u)$ | $x \rightarrow (u \rightarrow u_1)$ | $\overline{x}\,\overline{u}\,u_1$ |
| $\mathsf{LD}(u)$ | $\overline{x} \rightarrow (u \rightarrow u_0)$ | $x\,\overline{u}\,u_0$ |
| $\mathsf{HU}(u)$ | $x \rightarrow (u_1 \rightarrow u)$ | $\overline{x}\,\overline{u}_1\,u$ |
| $\mathsf{LU}(u)$ | $\overline{x} \rightarrow (u_0 \rightarrow u)$ | $x\,\overline{u}_0\,u$ |

The names for these clauses combine an indication of whether they correspond to variable $x$ being 1 ($\mathsf{H}$) or 0 ($\mathsf{L}$) and whether they form an implication from the node down to its child ($\mathsf{D}$) or from the child up to its parent ($\mathsf{U}$). When either node $u_0$ or $u_1$ is a leaf node, some of these clauses degenerate to tautologies. Such clauses are omitted from the proof. Each clause is numbered according to its position in the sequence of clauses comprising the proof. These defining clauses encode the assertion $u \leftrightarrow \mathit{ITE}(x, u_1, u_0)$, where *ITE* denotes the *if-then-else* operation, defined as $\mathit{ITE}(x, y, z) = (x \wedge y) \vee (\overline{x} \wedge z)$. As can be seen, the defining clauses are blocked with respect to extension variable $u$.

### 3.1 Generating BDD Representations of Clauses

The BDD representation $u$ of a clause $C$ is generated by using the ApplyOr operation on the BDD representations of its literals. This BDD has a simple, linear structure with one node for each literal. Each successive node has a branch to leaf node $L_1$ when the literal is true and to the next node in the chain when the literal is false. The proof that $C \vdash u$ is based on this linear structure, employing the upward defining clauses $\mathsf{HU}$ and $\mathsf{LU}$ for the nodes in the chain [28].

### 3.2 The ApplyAnd Operation

The key idea in generating proofs for the And operation is to follow the recursive structure of the Apply algorithm. We do this by integrating proof generation into the

| Terminal Cases | |
|---|---|
| Case | Result |
| $u = v$ | $(u, \top)$ |
| $u = L_0$ | $(L_0, \top)$ |
| $v = L_0$ | $(L_0, \top)$ |
| $u = L_1$ | $(v, \top)$ |
| $v = L_1$ | $(u, \top)$ |

```
ApplyAndRecur(u, v)
    J ⟵ {}
    x ⟵ min(Var(u), Var(v))
    if x = Var(u):
            u1, u0 ⟵ Hi(u), Lo(u)
            J ⟵ J ∪ {HD(u), LD(u)}
    else:   u1, u0 ⟵ u, u
    if x = Var(v):
            v1, v0 ⟵ Hi(v), Lo(v)
            J ⟵ J ∪ {HD(v), LD(v)}
    else:   v1, v0 ⟵ v, v
    w1, s1 ⟵ ApplyAnd(u1, v1)
    w0, s0 ⟵ ApplyAnd(u0, v0)
    J ⟵ J ∪ {s1, s0}
    if w1 = w0:
            w ⟵ w1
    else:
            w ⟵ GetNode(x, w1, w0)
            J ⟵ J ∪ {HU(w), LU(w)}
    s ⟵ JustifyAnd(⟨u, v, w⟩, J)
    AndCache(⟨u, v⟩) ⟵ (w, s)
    return (w, s)
```

**Fig. 1.** Terminal cases and recursive step of ApplyAnd operation, modified for proof generation. Each call returns both a node and a proof step.

ApplyAnd procedure. The overall control flow is identical to the standard version, except the function returns both a BDD node $w$ and a step number $s$. For arguments $u$ and $v$, the generated step $s$ has clause $\overline{u}\,\overline{v}\,w$ along with antecedents defining a resolution proof of the implication $u \wedge v \rightarrow w$. We refer to this as the *justification* for the operation. The operation cache is modified to hold both the returned node and the justifying step number as values.

Figure 1 shows the main components of the implementation. When the two arguments are equal or one of the leaves is a terminal node, then the recursion terminates (left). These cases have tautologies as their justification. Failing a terminal case, the code checks in the operation cache for matching arguments $u$ and $v$, returning the cached result if found.

Failing the terminal case tests and the cache lookup, the program proceeds as shown in the procedure ApplyAndRecur (right). Here, the procedure branches on the variable $x$ that is the minimum of the two root variables. The procedure accumulates a set of steps $J$ to be used in the implication proof. These include the two steps (possibly tautologies) from the two recursive calls. At the end, it invokes a function JustifyAnd to generate the required proof. It stores both the result node $w$ and the proof step $s$ in the operation cache, and it provides these values as the return values.

**Proof Generation for the General Case.** Proving the nodes generated by ApplyAnd satisfy the implication property proceeds by inducting on the structure of the argument

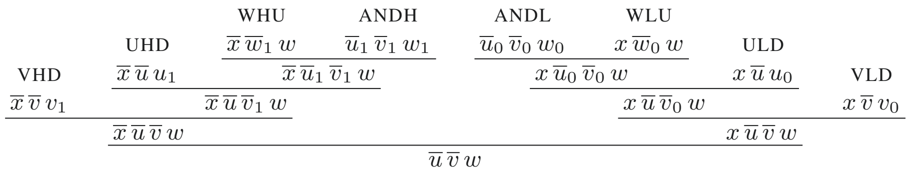

**Fig. 2.** Resolution proof for general step of the APPLYAND operation and result BDDs. That is, it can assume that the results $w_1$ and $w_0$ of the recursive calls to arguments $u_1$ and $v_1$ and to $u_0$ and $v_0$ satisfy the implications $u_1 \wedge v_1 \rightarrow w_1$ and $u_0 \wedge v_0 \rightarrow w_0$, and that these calls generated proof steps $s_1$ and $s_0$ justifying these implications. Figure 2 shows the structure of the resolution proof for the general case, where none of the equalities hold and the recursive calls do not yield tautologies. The proof relies on the following clauses as antecedents, arising from the recursive calls and from the defining clauses for nodes $u$, $v$, and $w$:

| Term | Formula | Clause | Term | Formula | Clause |
|---|---|---|---|---|---|
| ANDH | $u_1 \wedge v_1 \rightarrow w_1$ | $\overline{u}_1\, \overline{v}_1\, w_1$ | ANDL | $u_0 \wedge v_0 \rightarrow w_0$ | $\overline{u}_0\, \overline{v}_0\, w_0$ |
| WHU | $x \rightarrow (w_1 \rightarrow w)$ | $\overline{x}\, \overline{w}_1\, w$ | WLU | $\overline{x} \rightarrow (w_0 \rightarrow w)$ | $x\, \overline{w}_0\, w$ |
| UHD | $x \rightarrow (u \rightarrow u_1)$ | $\overline{x}\, \overline{u}\, u_1$ | ULD | $\overline{x} \rightarrow (u \rightarrow u_0)$ | $x\, \overline{u}\, u_0$ |
| VHD | $x \rightarrow (v \rightarrow v_1)$ | $\overline{x}\, \overline{v}\, v_1$ | VLD | $\overline{x} \rightarrow (v \rightarrow v_0)$ | $x\, \overline{v}\, v_0$ |

Along the left, the clauses cover the case of $x = 1$, first resolving clause ANDH and WHU, then resolving the result first with clause UHD and then clause VHD. A similar progression occurs along the right covering the case of $x = 0$. The two chains are then merged by resolving on variable $x$ to yield the final implication. As this figure illustrates, a total of seven resolution steps are required. These can be merged into two linear resolution chains, and so the proof generator produces at most two clauses per APPLYAND operation.

**Proof Generation for Special Cases.** The proof structure shown in Figure 2 only holds for the most general form of the recursion. However, there are many special cases, such as when the recursive calls yield tautologous results, when some of the child nodes are equal, and when the two recursive calls return the same node.

Our method for handling both the general and special cases relies on the V-shaped structure of the proofs, as is illustrated in Figure 2. That is, there are two linear chains, one along the left and one along the right consisting of some subsequence of the following clauses:

$$A_H = \text{ANDH}, \text{WHU}, \text{UHD}, \text{VHD}$$
$$A_L = \text{ANDL}, \text{WLU}, \text{ULD}, \text{VLD}$$

These will be proper subsequences when some of the clauses are not included in the set $J$ in APPLYAND (Figure 1), or they are tautologies. In addition, some of the clauses may be extraneous and therefore must not occur as antecedents.

Rather than trying to enumerate the special cases, we found it better to create a general-purpose linear chain resolver that handles all of the cases in a uniform way. This resolver is called on the each of the clause sequences $A_H$ and $A_L$. It proceeds through a sequence of clauses, discarding any tautologies and any clauses that do not resolve with the result so far. It then emits the proof clauses with the selected antecedents.

### 3.3 Testing Implication

| Terminal Cases | |
|---|---|
| Case | Result |
| $u = v$ | $\top$ |
| $u = L_0$ | $\top$ |
| $v = L_1$ | $\top$ |
| $u = L_1, v \neq L_1$ | Error |
| $v = L_0, u \neq L_0$ | Error |

PROVEIMPLICATIONRECUR$(u, v)$
- $J \longleftarrow \{\}$
- $x \longleftarrow \min(\mathsf{Var}(u), \mathsf{Var}(v))$
- **if** $x = \mathsf{Var}(u)$:
  - $u_1, u_0 \longleftarrow \mathsf{Hi}(u), \mathsf{Lo}(u)$
  - $J \longleftarrow J \cup \{\mathsf{HD}(u), \mathsf{LD}(u)\}$
- **else**: $u_1, u_0 \longleftarrow u, u$
- **if** $x = \mathsf{Var}(v)$:
  - $v_1, v_0 \longleftarrow \mathsf{Hi}(v), \mathsf{Lo}(v)$
  - $J \longleftarrow J \cup \{\mathsf{HU}(v), \mathsf{LU}(v)\}$
- **else**: $v_1, v_0 \longleftarrow v, v$
- $s_1 \longleftarrow$ PROVEIMPLICATION$(u_1, v_1)$
- $s_0 \longleftarrow$ PROVEIMPLICATION$(u_0, v_0)$
- $J \longleftarrow J \cup \{s_1, s_0\}$
- $s \longleftarrow$ JUSTIFYIMPLICATION$(\langle u, v\rangle, J)$
- *ImplyCache*$(\langle u, v\rangle) \longleftarrow s$
- **return** $s$

**Fig. 3.** Terminal cases and recursive step of PROVEIMPLICATION operation

When the existential quantification operation applied to node $u$ generates node $v$, the program generates a proof that $u \rightarrow v$, by calling procedure PROVEIMPLICATION with $u$ and $v$ as arguments. This procedure has the same recursive structure as the Apply algorithm, except that it does not generate any new nodes. It only returns the step number for a proof of the clause $\overline{u}\,v$. It uses an operation cache, but only to hold proof step numbers. Figure 3 shows the terminal cases for this procedure, as well as the recursion that occurs when neither a terminal case applies nor are the arguments found in the operation cache. A failure of the implication test indicates an error in the solver, and so it signals a fatal error if the implication does not hold.

Each recursive step accumulates up to six proof steps as the set $J$ to be used in the implication proof:

| Term | Formula | Clause | Term | Formula | Clause |
|---|---|---|---|---|---|
| IMH | $u_1 \rightarrow v_1$ | $\overline{u}_1\, v_1$ | IML | $u_0 \rightarrow v_0$ | $\overline{u}_0\, v_0$ |
| UHD | $x \rightarrow (u \rightarrow u_1)$ | $\overline{x}\,\overline{u}\,u_1$ | ULD | $\overline{x} \rightarrow (u \rightarrow u_0)$ | $x\,\overline{u}\,u_0$ |
| VHU | $x \rightarrow (v_1 \rightarrow v)$ | $\overline{x}\,\overline{v}_1\, v$ | VLU | $\overline{x} \rightarrow (v_0 \rightarrow v)$ | $x\,\overline{v}_0\, v$ |

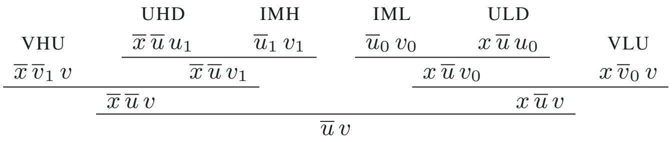

**Fig. 4.** Resolution proof for general step of the ProveImplication operation

The resolution proof for the general case is shown in Figure 4. It has a similar structure to the proof for the ApplyAnd operation, with two linear chains combined by a resolution on variable $x$. Our same general-purpose linear chain resolver can handle both the general case and the many special cases that arise.

## 4 Experimental Results

We implemented the proof-generating, SAT solver PGBDD (for Proof-Generating BDD). It is written entirely in Python and consists of around 2000 lines of code, including a BDD package, support for generating extended-resolution proofs, and the overall SAT solver framework.[1]

Although slow, it can handle large enough benchmarks to provide useful insights into the potential for a BDD-based SAT solver to generate proofs of challenging problems, especially when quantification is supported. It generates proofs in the LRAT format [9].

Our BDD package supports mark-and-sweep garbage collection. It starts the marking using the root nodes for all active terms in the sequence of root nodes $u_1, u_2, \ldots$. Following the marking phase, it traverses the unique table and eliminates the unmarked nodes. It also traverses the operation caches and eliminates any entries for which one of the argument nodes or the result node is unmarked. When a node is deleted, the solver can also direct the proof checker to delete its defining clauses. Similarly, when an entry is deleted from the operation cache, the solver can direct the proof checker to delete those clauses added while generating the justification for the entry.

In addition to the input CNF file, the program can accept a variable-ordering file, mapping the input variables in the CNF to their levels in the BDD.

The solver supports three different evaluation mechanisms:

**Linear:** Form the conjunction of the clauses, according to their order in the input file. No quantification is performed. This matches the operation described in [28].

**Bucket Elimination:** Place the BDDs representing the clauses into buckets according to the level of their topmost variable. Then process the buckets from lowest to highest. While a bucket has more than one element, repeatedly remove two elements, form their conjunction, and place the result in the bucket designated by the topmost variable. Once the bucket has a single element, existentially quantify the topmost

[1] The solver, along with code for generating and testing a set of benchmarks, is available at https://github.com/rebryant/pgbdd-artifact.

variable and place the result in the appropriate bucket [12]. This matches the operation described in [21].

**Scheduled:** Perform operations as specified by a scheduling file. This file contains a sequence of lines, each providing a command in a simple, stack-based notation:

| | |
|---|---|
| `c` $c_1, \ldots, c_k$ | Generate and push the BDDs for the specified clauses. |
| `a` $m$ | Pop and conjoin the top $m$ elements. Push the result. |
| `q` $v_1, \ldots, v_k$ | Quantify the top element by the specified variables. |

In generating benchmarks, we wrote programs to generate the CNF files, the variable orderings, and the schedules in a unified framework.

For all of our benchmarks we report the total number of clauses in the proof, including the input clauses, the defining clauses for the extension variables (up to four per BDD node generated) and the derived clauses (one per input clause and up to two per result inserted into either *AndCache* or *ImplyCache*.)

We compare the performance of our BDD-based SAT solver with that of KISSAT, the winner of the 2020 SAT competition [3], representing the state of the art in search-based SAT solvers.

### 4.1 Mutilated Chessboard

The mutilated chessboard problem considers an $n \times n$ chessboard, with the corners on the upper left and the lower right removed. It attempts to tile the board with dominos, with each domino covering two squares. Since the two removed squares had the same color, and each domino covers one white and one black square, no tiling is possible. This problem has been well studied in the context of resolution proofs, for which it can be shown that any proof must be of exponential size [1].

A standard CNF encoding involves defining Boolean variables to represent the boundaries between adjacent squares, set to 1 when a domino spans the two squares, and set to 0 otherwise. The clauses then encode an Exactly1 constraint for each square, requiring each square to share a domino with exactly one of its neighbors. We label the variables representing a horizontal boundary between a square and the one below as $y_{i,j}$, with $1 \leq i < n$ and $1 \leq j \leq n$. The variables representing the vertical boundaries are labeled $x_{i,j}$, with $1 \leq i \leq n$ and $1 \leq j < n$. With a mutilated chessboard, we have $y_{1,1} = x_{1,1} = y_{n-1,n} = x_{n,n-1} = 0$.

As the log-log plot in Figure 5 shows, PGBDD has exponential performance when using linear conjunction or bucket elimination. Indeed, KISSAT outperforms PGBDD when operating in these modes. However, KISSAT can also be seen to have exponential performance—to reach $n = 22$, it generates a proof with over 136 million clauses.

On the other hand, another approach, inspired by symbolic model checking [7] yields polynomial performance. It is based on the following observation: when processing the columns from left to right, the only information required to place dominos in column $j$ is the identity of those rows $i$ for which a domino crosses horizontally from $j-1$ to $j$. This information is encoded in the values of $x_{i,j-1}$ for $1 \leq i \leq n$.

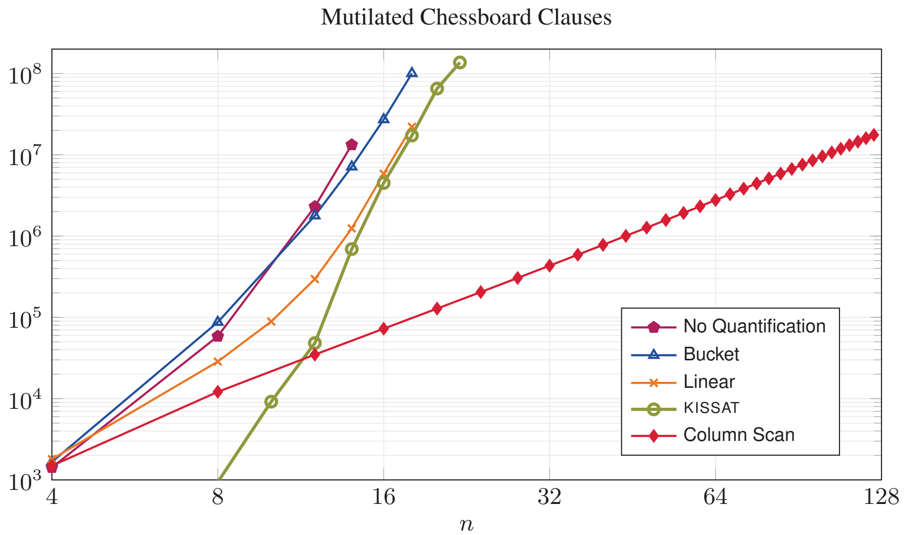

**Fig. 5.** Total number of clauses in proofs of $n \times n$ mutilated chess boards. The proofs using the column scanning approach grow as $n^{2.69}$.

Let us group the variables into columns, with $X_j$ denoting variables $x_{1,j}, \ldots, x_{n,j}$, and $Y_j$ denoting variables $y_{1,j}, \ldots, y_{n-1,j}$. Scanning the board from left to right, consider $X_j$ to encode the "state" of processing after completing column $j$. As the scanning process reaches column $j$, there is a *characteristic function* $\sigma_{j-1}(X_{j-1})$ describing the set of allowed crossings of horizontally-oriented dominos from column $j-1$ into column $j$. No other information about the configuration of the board to the left is required. The characteristic function after column $j$ can then be computed as:

$$\sigma_j(X_j) = \exists X_{j-1} \left[\sigma_{j-1}(X_{j-1}) \land \exists Y_j \; T_j(X_{j-1}, Y_j, X_j)\right] \qquad (1)$$

where $T_j(X_{j-1}, Y_j, X_j)$ is a "transition relation" consisting of the conjunction of the Exactly1 constraints for column $j$. From this, we can existentially quantify the variables $Y_j$ to obtain a BDD encoding all compatible combinations of the variables $X_{j-1}$ and $X_j$. By conjuncting this with the characteristic function for column $j-1$ and existentially quantifying the variables $X_{j-1}$, we obtain the characteristic function for column $j$. With a mutilated chessboard, we generate leaf node $L_0$ in attempting the final conjunction. Note that Equation (1) does not represent a reformulation of the mutilated chessboard problem. It simply defines a way to schedule the conjunction and quantification operations over the input clauses and variables.

In our experiments, we found that this scanning reaches a fixed point after processing $n/2$ columns. That is, from that column onward, the characteristic functions become identical, except for a renaming of variables. This indicates that the set of all possible horizontal configurations stabilizes halfway across the board. Moreover, the BDD representations of the states grow as $O(n^2)$. For $n = 124$, the largest has just 3,969 nodes.

One important rule-of-thumb in symbolic model checking is that the successive values of the next-state variables must be adjacent in the variable ordering. Furthermore, the vertical variables in $Y_j$ must be close to their counterparts in $X_{j-1}$ and $X_j$. Both objectives can be achieved by ordering the variables row-wise, interleaving the variables $x_{i,j}$ and $y_{i,j}$, ordering first by row index $i$ and then by column index $j$. This requires the quantification operations of Equation 1 to be performed on non-root variables.

Figure 5 shows that the "column-scanning" approach yields performance scaling as $n^{2.69}$, allowing us to handle cases up to $n = 124$. Keep in mind that the problem size here should be measured as $n^2$, the number of squares in the board. Thus, a problem instance with $n = 124$ is over 31 times larger than one with $n = 22$ (the upper limit reached by KISSAT), in terms of the number of input variables and clauses. Indeed, the case of $n = 22$ is straightforward for PGBDD, requiring only a few seconds and generating a proof with 161,694 clauses.[2] By contrast, KISSAT requires 12.6 hours and generates over 136 million clauses.

The plot labeled "No Quantification" demonstrates the importance of including existential quantification in solving this problem. These data were generated by using the same schedule as with column scanning, but with all quantification operations omitted. As can be seen, this approach could not scale beyond $n = 14$.

Most attempts to generate propositional proofs of the mutilated chessboard have exponential performance. No solver in the 2018 SAT competition could handle the instance with $n = 20$. Heule, Kiesl, and Biere [19] devised a problem-specific approach that could generate proofs up to $n = 50$ by exploiting special symmetries in the problem, using a set of rewriting rules to dramatically reduce the search space. Our approach also exploits symmetries in the problem, but by exploiting a way to compactly encode the set of possible configurations between successive columns. Other than these two, we know of no other approach for generating polynomially-sized propositional proofs for the problem.

### 4.2 Pigeonhole Problem

The pigeonhole problem is one of the most studied problems in propositional reasoning. Given a set of $n$ holes and a set of $n+1$ pigeons, it asks whether there is an assignment of pigeons to holes such that 1) every pigeon is in some hole, and 2) every hole contains at most one pigeon. The answer is no, of course, but any resolution proof for this must be of exponential length [15]. Groote and Zantema have shown that any BDD-based proof of the principle that only uses the Apply algorithm must be of exponential size [14]. On the other hand, Cook constructed an extended resolution proof of size $O(n^4)$, in part to demonstrate the expressive power of extended resolution [8].

We consider two encodings of the problem. Both are based on a set of variables $p_{i,j}$ for $1 \leq i \leq n$ and $1 \leq j \leq n+1$, with the interpretation that pigeon $j$ is assigned to hole $i$. Encoding the property that each pigeon $j$ is assigned to some hole can be expressed as a single clause:

$$Pigeon_j = \bigvee_{i=1}^{n} p_{i,j}$$

[2] All times reported here were measured on a 3 GHz Intel i7-9700 CPU with 16GB of memory.

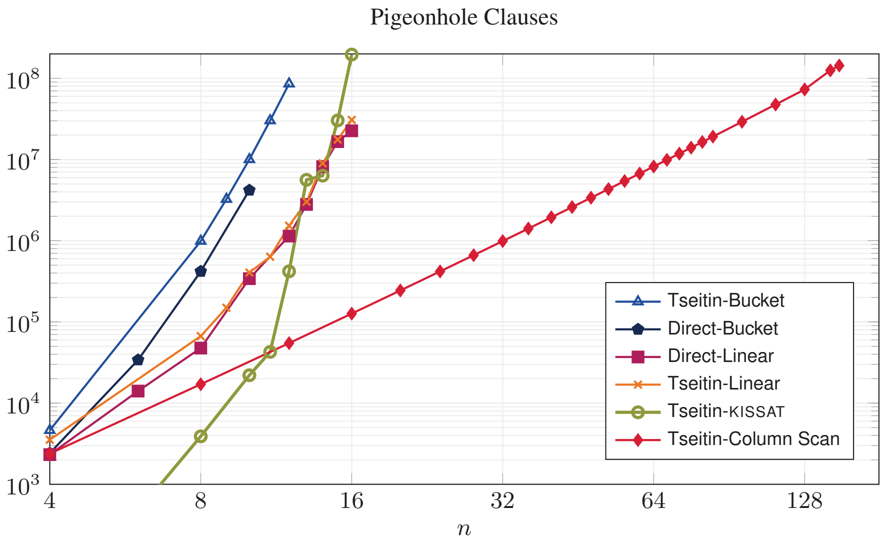

**Fig. 6.** Total number of clauses in proofs of pigeonhole problem for $n$ holes. Using a direct encoding led to exponential performance, but using a Tseitin encoding and column scanning gives proofs that grow as $n^{3.03}$.

Encoding the property that each hole $i$ contains at most one pigeon can be done in two different ways. A *direct* encoding simply states that for any pair of pigeons $j$ and $k$, at least one of them must not be in hole $i$:

$$Direct_i = \bigwedge_{j=1}^{n+1} \bigwedge_{k=j+1}^{n+1} \overline{p}_{i,j} \vee \overline{p}_{i,k}$$

This encoding requires $\Theta(n^2)$ clauses for each hole, yielding a total CNF size of $\Theta(n^3)$.

A second, *Tseitin* encoding introduces Tseitin variables to track which holes are occupied, starting with pigeon 1 and working upward. We use an encoding published by Sinz [27] that uses Tseitin variables $s_{i,j}$ for $1 \leq i \leq n$ and $1 \leq j \leq n$, where $s_{i,j}$ equals 1 if a pigeon $j'$ occupies hole $i$ for some $j' \leq j$. It requires $3n - 1$ clauses and $n$ Tseitin variables per hole, yielding an overall CNF size of $\Theta(n^2)$.

As is illustrated by the log-log plots of Figure 6, this choice of encoding not only affects the CNF size, it dramatically affects the size of the proofs generated by PGBDD. With a direct encoding, we could not find any combination of evaluation strategy or variable ordering that could go beyond $n = 16$. Similarly, the Tseitin encoding did not help when using linear evaluation or bucket elimination. Indeed, we see KISSAT, using the Tseitin encoding, matching or exceeding our program for these cases, but all of these have exponential performance. (KISSAT could only reach $n = 15$ when using a direct encoding.)

On the other hand, the column scanning approach used for the mutilated checkerboard can also be applied to the pigeonhole problem when the Tseitin encoding is used. Consider an array with hole $i$ represented by row $i$ and pigeon $j$ represented by column $j$. Let $S_j$ represent the Tseitin variables $s_{i,j}$ for $1 \leq i \leq n$. The "state" is then encoded in these Tseitin variables. In processing pigeon $j$, we can assume that the possible combinations of values of Tseitin variables $S_{j-1}$ is encoded by a characteristic function $\sigma_{j-1}(S_{j-1})$. In addition, we incorporate into this characteristic function the requirement that each pigeon $k$, for $1 \leq k \leq j-1$ is assigned to some hole. Letting $P_j$ denote the variables $p_{i,j}$ for $1 \leq i \leq n$, the characteristic function at column $j$ can then be expressed as:

$$\sigma_j(S_j) = \exists S_{j-1} \left[\sigma_{j-1}(S_{j-1}) \wedge \exists P_j \; T_j(S_{j-1}, P_j, S_j)\right] \quad (2)$$

where the "transition relation" $T_j$ consists of the clauses associated with the Tseitin variables, plus the clause encoding constraint $\mathit{Pigeon}_j$. As with the mutilated chessboard, having a proper variable ordering is critical to the success of a column scanning approach. We interleave the ordering of the variables $p_{i,j}$ and $s_{i,j}$, ordering them first by $i$ (holes) and then by $j$ (pigeons.)

Figure 6 demonstrates the effectiveness of the column-scanning approach. We were able to handle instances up to $n = 150$, and with an overall performance trend of $n^{3.03}$. Our achieved performance therefore improves on Cook's bound of $O(n^4)$. A SAT-solving method developed by Heule, Kiesl, Seidl, and Biere can generate short proofs of multiple encodings of pigeon hole formulas, including the direct encoding [20]. These proofs are similar to ours after transforming them into the same proof format and the size is also $O(n^3)$ [17].

Unlike with the mutilated chessboard, the scanning does not reach a fixed point. Instead, the BDDs start very small, because they must encode the locations of only a small number of occupied holes. They reach their maximum size at pigeon $n/2$, as the number of combinations for occupied and unoccupied holes reaches its maximum. Then the BDD sizes drop off as the encoding needs to track the positions of a decreasing number of unoccupied holes. Fortunately, all of these BDDs grow quadratically with $n$, reaching a maximum of 5,702 nodes for $n = 150$.

### 4.3 Evaluation

Overall, our results demonstrate the potential for generating small proofs of unsatisfiability using BDDs. We have achieved polynomial performance for problems for which search-based SAT solvers have exponential performance.

Other studies have compared BDDs to search-based SAT on a variety of benchmark problems. Several of these observed exponential performance for BDD-based solvers for problems for which we have obtained polynomial performance. Uribe and Stickel [31] ran experiments with the mutilated chessboard problem, but they did not do any variable quantification. Pan and Vardi [25] applied a variety of scheduling and variable ordering strategies for the mutilated chessboard and pigeonhole problems. Although they were able to get better performance than with a search-based SAT solver, they still observed exponential scaling. Obtaining polynomial performance for these problems requires more problem-specific approaches than the ones they considered.

Table 1 provides some performance data for the largest instances solved for the two benchmark problems. A first observation is that these problems are very large, with tens of thousands of input variables and clauses.

**Table 1.** Summary data for the largest problems solved

| Instance | Chessboard Chess-124 | Pigeonhole Pigeon-Tseitin-150 |
|---|---|---|
| Input variables | 30,500 | 45,150 |
| Total BDD nodes | 3,409,112 | 17,861,833 |
| Maximum live nodes | 198,967 | 225,446 |
| Input clauses | 106,136 | 67,501 |
| Defining clauses | 12,127,031 | 62,585,397 |
| Derived clauses | 5,348,303 | 81,019,084 |
| Maximum live clauses | 751,944 | 1,297,039 |
| SAT time (secs) | 5,366 | 5,206 |
| Checking time (secs) | 30 | 240 |

The total number of BDD nodes indicates the total number generated by the function GETNODE, and for which extension variables are created. These are numbered in the millions, and far exceed the number of input variables. On the other hand, the maximum number of live nodes shows the effectiveness of garbage collection—at any given point in the program, at most 6% of the total number of nodes must be stored in the unique table and tracked in the operation caches. Garbage collection also keeps the number of clauses that must be tracked by the proof checker below 5% of the total number of clauses. The elapsed time for the SAT solver ranges up to 1.5 hours. We believe, however, that an implementation in a more performant language would reduce these times greatly. The checking times are shown for an LRAT proof checker written in the C programming language. The proofs have also been checked with a formally verified proof checker based on the HOL theorem prover [29].

## 5 Conclusion

Biere, Sinz, and Jussila [21,28] made the critical link between BDDs and extended resolution proofs. We have shown that adding the ability to perform arbitrary existential quantification can greatly increase the performance of a proof-generating, BDD-based SAT solver.

Generating proofs for the two benchmarks problems required special insights into their structure and then crafting evaluation mechanisms to exploit their properties. We believe, however, that the column scanning approach we employed could be generalized and made more automatic.

The ability to generate correctness proofs in a BDD-based SAT solver invites us to consider generating proofs for other tasks to which BDDs are applied, including QBF solving, model checking, and model counting. Perhaps a proof of unsatisfiability could provide a useful building block for constructing correctness proofs for these other tasks.
